# Conjugated linoleic acid producing potential of lactobacilli isolated from goat (AXB) rumen fluid samples

**DOI:** 10.5713/ajas.19.0080

**Published:** 2019-08-23

**Authors:** Amrish Kumar Tyagi, Sachin Kumar, Prasanta Kumar Choudhury, Bhawna Tyagi, Nitin Tyagi

**Affiliations:** 1 Rumen Biotechnology Lab, Animal Nutrition Division, ICAR-National Dairy Research Institute, Karnal, Haryana 132001, India; 2 Gut Microbial Function, CSIRO Agriculture and Food, Queensland Bioscience Precinct, St Lucia, Brisbane 4067, Australia; 3 Dairy Technology Department, Centurion University of Technology and Management, Paralakhemundi-761211, Odisha, India

**Keywords:** Biohydrogenation, Conjugated Linoleic Acid, Goat, Lactobacilli, Rumen

## Abstract

**Objective:**

The present investigation was aimed to explore the potential of lactobacilli for conjugated linoleic acid (CLA) production, isolated from rumen fluid samples of lactating goats.

**Methods:**

A total of 64 isolates of lactobacilli were obtained using deMan-Rogosa-Sharpe (MRS) agar from rumen fluid of goats and further subjected to morphological and biochemical characterizations. Isolates found as gram-positive, catalase negative rods were presumptively identified as *Lactobacillus* species and further confirmed by genus specific polymerase chain reaction (PCR). The phylogenetic tree was constructed from the nucleotide sequences using MEGA6.

**Results:**

Out of the 64 isolates, 23 isolates were observed positive for CLA production by linoleate isomerase gene-based amplification and quantitatively by UV-spectrophotometric assay for the conversion of linoleic acid to CLA as well as gas chromatography-based assay. In all Lactobacillus species cis9, trans11 isomer was observed as the most predominant CLA isomer. These positive isolates were identified by 16S rRNA gene-based PCR sequencing and identified to be different species of *L. ingluviei* (2), *L.salivarius* (2), *L. curvatus* (15), and *L. sakei* (4).

**Conclusion:**

The findings of the present study concluded that lactic acid bacteria isolated from ruminal fluid samples of goat have the potential to produce bioactive CLA and may be applied as a direct fed microbial to enhance the nutraceutical value of animal food products.

## INTRODUCTION

Animal food and food products of ruminant origin are the major sources of nutrients, however, in some cases they are considered to be harmful to human health due to high risk for cardiovascular diseases as they have high level of saturated fatty acids (FA) and cholesterol [1]. Furthermore, ruminant fat both in milk and meat contains many specific FA such as vaccenic acid, n-3, n-6 poly unsaturated fatty acids and conjugated linoleic acid (CLA), which are associated with their various health promoting properties. The CLA is a mixture of 28 positional and geometric isomers of linoleic acid (LA) present in animal products having various nutraceutical characteristics [2–4]. It is formed mainly as an intermediate during the biohydrogenation of LA in the rumen [5] or from the endogenous conversion of *trans*-vaccenic acid in the mammary gland [6]. The potent biological effect of CLA such as anti-carcinogenic, anti-inflammatory, anti-obesity, anti-diabetic, immunomodulatory, anti-atherosclerotic, reduction of whole-body fat and bone formation promoting properties have been identified in a wide range of animal species [2,4,7]. The recommended dietary allowance of CLA for humans ranges from 1 to 3 g/d to accomplish desired health benefits [8]. However, the availability (36 to 440 mg/d) is very much lower than the requirement to exert the beneficial effects. Chemical synthesis by alkaline isomerization of LA rich oil to produce CLA is quite expensive and yields unenviable side products. Although ruminant derived products are the richest source of CLA in the human diet, still the concentration present in the foodstuffs is too low and depends upon feedstock and type of animal breed [9]. Therefore, there is an ever-increasing interest to augment this constituent in animal food products for better health aspects.

Dietary intervention and strategies for animal management have the possibility of creating rumen environment that alters FAs biohydrogenation and enhance CLA content in milk. Rumen microbial fermentation is found to improve the CLA concentration in animal products [10]. Hence, there is an opportunity to further increase the CLA content of animal products by employing these rumen microbes [11]. This approach can be feasible by adding the ruminant’s diet rich in LA and/or by supplementing the microbes directly into the rumen having potential of CLA production which ultimately lead to foods of animal origin with enhanced nutraceutical importance. A number of CLA producing bacteria of the rumen origin including *Butyrivibrio fibrisolvens* [12], lactic acid bacteria (LAB) namely *Lactobacillus acidophilus*, *Lactococcus lactis* subsp. *cremoris*, *L. lactis* subsp. *lactis*, *L. delbrueckii* subsp. *bulgaricus*, *L. delbrueckii* subsp. *Lactis* and have been reported to convert LA into CLA [11]. However, information is lacking regarding CLA production from *Lactobacillus* species of rumen origin of Indian breeds except few reports despite the well-known fact that LAB possess generally recognized as safe status and can be applied as probiotics in animal health improvement. The present study, therefore, was conducted to isolate, identify and characterize the CLA producing lactobacilli from rumen samples of AXB goats.

## MATERIALS AND METHODS

### Sampling and isolation of lactobacilli

The rumen sampling was performed from crossbreed lactating goats (Anglo Nubian×Beetal; age ~3 years; weight ~30 kg) maintained at Livestock Research Centre, ICAR-National Dairy Research Institute, Karnal-132001, Haryana. The goats were not fed with any probiotics. Around 100 mL of rumen fluid was collected using a suction pump and immediately transported to the laboratory. Samples were collected in O_2_ free CO_2_ flushed sterilized containers and homogenized before further processing. One mL of each sample was suspended in deMan-Rogosa-Sharpe (MRS) broth, containing LA (0.5 mg/mL) and incubated at 39°C for 24 h [13]. Samples were serially diluted in peptone water (0.1%) and subsequently plated on MRS agar with incubation up to 3 days at 39°C. Colonies were picked up randomly to MRS broth and the isolates were streaked on MRS agar plates for further purification. The purity of cultures was examined microscopically after performing Gram-staining and preserved at −80°C in glycerol stocks (10%). For routine use, the isolates were maintained in MRS broth at 4°C and freshly activated at each time before use.

### Genomic DNA isloation

Molecular confirmation of the isolates as lactobacilli was done after DNA isolation followed by polymerase chain reaction (PCR) amplification using genus specific primers. Genomic DNA was isolated as per the modified methods of Jena et al [14] from MRS broth of respective isolates. Briefly, the bacterial cell suspension was transferred to a 2 mL microcentrifuge tube and pelleted by centrifugation at 8,000×g for 10 min. Then 800 μL cetyl trimethyl ammonium bromide extraction buffer was added to the cells and mixed thoroughly. The cell suspension was incubated at 70°C for 1 h and vortexed every 15 min of interval. A volume of 500 μL of chloroform:isoamyl alcohol (24:1) was added to the suspended pellet and mixed upside down to form a white emulsion. In the next step, centrifugation was carried out at 8,000×g for 20 min. The aqueous layer was transferred, and DNA was precipitated with addition of 300 μL of isopropanol and centrifuged (8,000×g, 10 min). Subsequently, 500 μL of 70% ethanol was added to the white pellet with mixing. After centrifugation pellet was air dried and suspended in Tris-ethylenediaminetetraacetic acid (Tris-EDTA) buffer (pH 8.0) and incubated at 60°C for 10 min. The quality of isolated DNA was checked by 0.8% agarose gel electrophoresis and concentration was quantified by Nano drop plate reader (Tecan-Infinite Pro 200, Männedorf, Switzerland) and thereafter, stored at −20°C for further use.

### The genus level confirmation

For identification of bacterial isolates at genus level, PCR was performed with the following primer pairs and PCR conditions [15]. The PCR reaction mixture (50 μL) contained dNTPs each 2.5 mM; each primer 20 pmol; 10× PCR bufer 5 μL; *Taq* DNA polymerase (Genetix, New Delhi, India) 1 U and template DNA 100 ng. The forward primer was LbLMA1-rev (5′-CTCAAAACTAAACAAAGTTTC-3′) and the reverse primer was R16-1(5′-CTTGTACACACCGCCCGTCA-3′). PCR amplification was carried out in a thermal cycler (Quanta Biotech-96, Beverly, MA, USA) with initial denaturation of 94°C for 5 min followed by 35 cycles of denaturation at 94°C for 30 s, annealing at 55°C for 45 s and extension at 72°C for 30 s, followed by a final extension at 72°C for 7 min. Amplified DNA fragments were examined by horizontal agarose gel electrophoresis containing ethidium bromide (0.5 μg/mL) at 100 V for 1 h in 1× Tris-Borate-EDTA bufer with 5 μL aliquots of PCR products. The gel images were digitized through Genview (New Delhi, India), Genetix (India), Biotech Asia Pvt. Ltd. (New Delhi, India). Further, all the positive isolates were checked for PCR based linoleate isomerase (LAI) gene amplification.

### Linoleate isomerase gene amplification

The presence of the LAI gene responsible for the conversion of LA to CLA was checked by PCR based amplification as per the earlier methods [16]. Briefly, PCR amplification of target gene was carried using the following set of primers LISO3 - (5′-CGGACNTACGTYGAYTTAATGG-3′); and LISO4- (5′-TGGTGMACMACRATCGACAT-3′) containing a reaction volume of 25 μL (2.5 mM dNTPs each; each primer 10 pmol; 10× PCR bufer 2.5 μL; *Taq* DNA polymerase [Genetix, India] 0.5 U; 1 μL MgCl_2_ [20 mM] and template DNA 50 ng). PCR amplification was carried out in a thermal cycler (Quanta Biotech-96, USA) with initial denaturation of 94°C for 5 min followed by 35 cycles of denaturation at 94°C for 30 s, annealing at 56°C for 45 s and extension at 72°C for 1 min, followed by a final extension at 72°C for 5 min. Amplified DNA fragments were examined by horizontal agarose gel electrophoresis and gel images were digitized as described in previous section. All the positive isolates were further screened for CLA production assay from LA by spectrophotometric methods.

### UV-based spectrophotometric screening for conjugated linoleic acid production

After PCR confirmation lactobacilli isolates were further characterized for CLA biosynthesis in MRS broth using the UV-based spectrophotometric assay. A stock solution of LA (5 mg/mL, 99% purity; Sigma-Aldrich, St. Louis, MO, USA) was prepared in sterile distilled water with 1% (w/v) Tween-80 (Hi-media, Mumbai, India) and sterilised through a 0.2 μm syringe filter. For screening, the cultures were inoculated at 1% (v/v) to 10 mL MRS broth supplemented with 0.05% L-cys-HCl and 0.5 mg/mL of LA as a substrate and incubated at 37°C for 24 h. Subsequently, the lactobacilli isolates were tested for the efficacy to produce CLA in accordance to Barrett et al [17]. Briefly, the samples were centrifuged at 13,000 rpm at 4°C for 5 min, the supernatant (1 mL) was vigorously mixed with 2 mL of isopropanol and left undisturbed for 3 min. To this, 1.5 mL of hexane was added for extraction of FAs and remained undisturbed for 3 min. An aliquot 1 mL was taken for absorbance at 233 nm in UV-VIS spectrophotometer (Specord-200, Schönwalde-Glien, Germany). A standard curve (0.5 to 10 μg/mL) was prepared from reference *trans*10, *cis*12 CLA isomer to quantify total CLA and hexane layers containing only LA were used as blank in the entire assay.

### Fatty acid methyl esters synthesis and gas chromatographicanalysis

Fatty acid methyl esters (FAME) was prepared following direct synthesis method of O’Fallon et al [18] with little modifications. The FA analysis of MRS medium was performed directly on the liquid solutions. Lactobacilli-MRS medium was hydrolyzed for 1.5 h at 55°C in 10 N potassium hydroxide (KOH) in methanol. C^19:0^ in methanol was used as an internal standard. The KOH is neutralized, and the free FAs were methylated by sulfuric acid catalysis for 1.5 h at 55°C. Hexane was then added to the reaction tube, which was vortex-mixed and centrifuged. The hexane layers containing FAs were dried under a stream of N_2_ and re-dissolved in minimum volume of hexane. The samples were stored in glass vials at −20°C until analysed in gas chromatography (GC).

One μL sample (FAME) was injected into a fully automated Bruker 450 GC machine (Bruker Daltonik GmbH, Bremen, Germany) equipped with Rtx-2330 capillary column (120 m×0.25 mm I.D., 0.20 μm film thickness, Restek corporation, Bellefonte, PA, USA), an automated injector and a flame ionization detector in (1:50) split mode using hydrogen as a carrier gas. The temperatures of injector and detector were set at 260°C and 270°C, respectively. The temperature of column oven was programmed from 170°C to 240°C with step increase of 4°C/min. The quantitative analysis of CLA isomers was performed by comparison of retention times with methylated CLA standards (*cis*9, *trans*11; *trans*10, *cis*12 and *trans*9, *trans*11). The FAMEs were identified by comparing retention times with those of known standards. The areas of the individual peaks were used to determine the relative percentage of each FA present in the samples.

### 16S rRNA gene sequencing, identification and phylogeny

The DNA samples of the positive isolates confirmed for CLA production were further amplified with universal 16S rRNA gene primers; 27F 5′-AGAGTTTGATCCTGGCTCAG and 1492R 5′-GGTTACCTTGTTACGACTT [19]. The universal primers targeting partial 16S rRNA gene region were used to amplify the PCR reaction. The PCR components (10× PCR buffer; 10 μM each primer; 10 mM each dNTPs mix; *Taq* DNA polymerase 1 U; template DNA 100 ng) were mixed properly and the reaction was executed in a 50 μL of PCR reaction mixture under normal PCR cycling conditions for 35 cycles. The amplified PCR products were gel purified with QIA quick Gel extraction kit (Cat#28704) as per the manufacturer’s instructions (QIAGEN, Hilden, Germany) and out sourced for sequencing in both directions (Eurofins Genomics, India, Pvt. Ltd., Bengaluru, Karnataka, India). The sequences received from the chromatogram were retrieved and verified in BioEdit software and both strands were aligned with CLUSTAL W. Then the sequences were BLAST analysed to search for the sequence resemblances with other *Lactobacillus* sequences (http://blast.ncbi.nlm.nih.gov/) and were submitted to NCBI GenBank using BankIt submission tool. All the cultures were provided with accession numbers were recorded (MF148304-07 and MG430183-201). The phylogenetic tree was constructed from the nucleotide sequences using MEGA6 and 7 references sequences (MF992225, GU125609, LC 130553, KC416998, MG266175, FJ378897, AB911500, and MG430201) were included in this analysis.

### Biochemical characterization

Out of all the positive isolates, 19 isolates failed to differentiate on the basis of 16S rRNA gene sequence analysis, so further for species level confirmation biochemical tests viz. xylose and mellibiose fermentation and arginine hydrolysis was executed in basal medium (MRS medium without carbohydrate source) supplemented with 1% of each substrate.

## RESULTS AND DISCUSSION

### Isolation and confirmation of conjugated linoleic acid producing *Lactobacillus* spp

**Figure 1:**
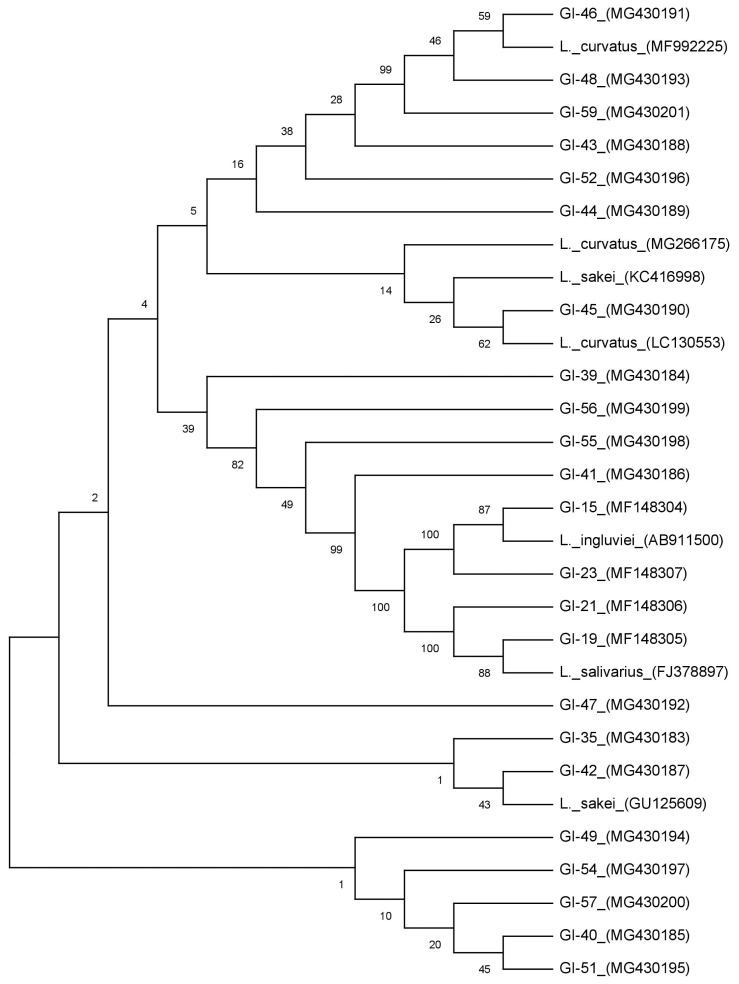
Evolutionary relationships of isolated conjugated linoleic acid producing *Lactobacillus* species isolated from rumen samples of goat by phylogenetic tree constructed from all the sequences.

A total of 64 isolates were isolated on selective media (MRS Agar) supplemented with LA from rumen fluid content of goats. Rice straw shaped colonies were observed morphologically on MRS agar plates and microscopically isolates were found to be Gram positive rods with negative catalase and oxidase activity, a common characteristic of *Lactobacillus* (Table 1). These isolates were designated as goat isolates (GI) followed by isolate number (Figure 1). Molecular level confirmation based on genus specific PCR amplification showed a product size of approximately 220 bp with all the isolates. The similar set of primers (LbLMA1 and rev/R16-1) was also used by many researchers to confirm *Lactobacillus* spp. coding for a spacer region between the 16S and 23S rRNA genes [13]. These positive isolates confirmed at genus level were further observed for LAI gene-based PCR amplification. In a number of studies, it was reported LAI gene to be a multi-component enzyme system, encoded in the genome of lactobacilli responsible for the biohydrogenation of LA to CLA with various intermediate steps [2,16,20]. Out of 64 isolates only 23 isolates showed a positive PCR amplification with desired product size around 968 bp.

### Conjugated linoleic acid production in MRS broth supplemented with linoleic acid

**Figure 2:**
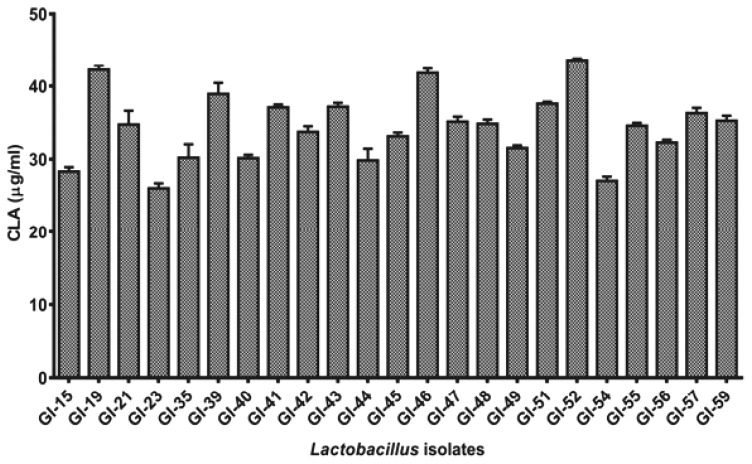
Comparative conjugated linoleic acid production (in μg/mL) assay of the positive *Lactobacillus* isolates after 24 h incubation.

All the 23 positive isolates were further studied quantitatively for the production of CLA in MRS broth supplemented with LA with active cultures of these isolates by UV-spectrophotometric based assay [17]. After 24 h of conversion, all the isolates were observed to produce 26.03 to 43.62 μg/mL of CLA from LA (Figure 2). Highest production of CLA was observed with GI-52 while lowest production was recorded from the isolate GI-23. Only 3 isolates (GI-19, GI-46, and GI-52) showed more than 40 μg/mL of CLA and most of the isolates showed an average 30 μg/mL of CLA in MRS supplemented broth.

Although the accurate pathway of CLA synthesis is still obscure, nevertheless, it has been observed that a LA detoxifying mechanism is responsible to eradicate the toxic effects of the substrate for cell survival as the assimilation of LA into bacterial cell membrane changes the membrane potential, lipid bilayer chemistry and intra-membrane machinery [9]. Highest CLA production (95.25 μg/mL) has been observed in *Lactobacillus plantarum* (*L. plantarum*) amongst lactobacilli strains by Khosravi et al [21]. Kishino et al [22] reported the potential of lactobacilli strains with high CLA production ranging from 3.41 mg/mL to 0.07 mg/mL. Our results and variation of CLA are in concurrence with observations of earlier studies of LAB isolates [23]. Six isolates of *L. plantarum* with minor variations in CLA conversions from 3.85% to 4.90% in traditional dairy origin was also reported [24]. The results strongly support with the fact that strain variation in lactobacilli has different ability to release CLA from LA. In our study, though higher production of CLA was observed, the source of isolation and strain variation may have higher degree of CLA producing capabilities.

### Gas chromatographic analysis of conjugated linoleic acid isomers

Health beneficial effects of CLA are specific to their respective isomers, hence proper analysis is necessary prior to use. All the 23 lactobacilli isolates having CLA production were studied for specific isomer production in MRS medium supplemented with desired LA. Out of all the lactobacilli strains 12 isolates were observed to produce c9,t11; t10,c12; and t9,t11 CLA isomers in the mediums. In all 23 lactobacilli c9,t11 isomer was observed as the most predominant isomer. The observation of the current study is in agreement with previous findings of isomer variability [9,11,23]. Strains GI-15, GI-35, GI-40, GI-44, and GI-54 did not show the production of t9, t11 isomers, whereas, few strains (GI-45, GI-49, and GI-56) showed the presence of c9,t11 and t9,t11 isomers and no t10, c12 isomer production. The findings are also substantiated by earlier observations that biosynthesis of t9,t11 CLA isomer was a biotransformation consequence of c9,t11 CLA [13].

The observations of this study indicated that synthesis isomers of CLA are strain dependent. Lee et al [25] reported production of c9, t11 (26.8 μg/mL) and t10, c12 (6.4 μg/mL) isomers from *L. plantarum* PL62 isolated from infant faces. Eight different CLA isomers with the enzyme extract of *L. acidophilus* CCRC 14079 with LA (t8,t10; t9,t11; t10,t12; t11,t13; t8,c10; c9,t11; t10,c12; and c11,t13) have been reported by Lin et al [26]. In connection to these findings, Sosa-Castañeda et al [9] assessed the CLA production potential of 13 strains of lactobacilli isolates and reported *L. fermentum* J20 to produce c9, t11 (42.63 μg/mL) and t10,c12 (8.27 μg/mL) CLA isomers. In another study, *L. plantarum* JCM 1551 was reported to produce 2.4 μg/mL of CLA, comprised of c9,t11 (21%) and t9, t11 (79%) CLA isomers [27] which are in contrast to our observations. The biotransformation of LA to CLA isomers is an isomerization process of linoleate isomerase enzyme [20] that is a multi-component enzymatic system responsible for biohydrogenation activity. The variability of isomers observed in CLA production supported varying capability of strains of different isomeric forms of linoleate isomerase enzyme [28]. In overall, isolate GI-52 was observed to be the most competent producer of CLA in terms total CLA production.

### Identification of the conjugated linoleic acid positive isolates

All the positive isolates were 16S rRNAgene amplified and showed a product size of approximately 1,500 bp. BLAST analysis revealed more than 99% homology with existing sequences. Phylogenetic tree constructed from all the sequences differentiated the isolates into 3 different clusters (Figure 1). Out of 23 isolates Cluster-I comprises of 19 isolates, whereas Cluster-II and III comprises 2 isolates in each group respectively. In Cluster-I all the isolates grouped together with reference sequences of *Lactobacillus curvatus* (*L. curvatus*) and *Lactobacillus sakei* (*L. sakei*) and could not differentiate into separate groups with a similarity of more than 99% homology. These results are in agreement with many findings, where it was reported that it is hard to discriminate *L. curvatus* and *L. sakei* as the genetic heterogeneity within the species is nearly similar [29,30]. Based on phenotypic and genotypic properties [31], primers developed from random amplified polymorphic DNA fingerprints and multiplex PCR-based analysis [30], were used to differentiate in between these species. Hence, based on melibiose fermentation and ammonia liberation from arginine hydrolysis, the isolates were differentiated into *L. curvatus* and *L. sakei* (Table 1). In Cluster-II, 2 of our isolates were grouped with *Lactobacillus salivarius* (*L. salivarius*), whereas Cluster-III isolates (2) showed similarity with L*actobacillus ingluviei* (*L. ingluviei*) (Figure 1).

Earlier reports have showed the production of CLA by LAB [21]. Lower production of CLA was reported by strains of *L. curvatus* and *L. sakei* (4.2% and 1.6%, respectively), as meat fermentation starter cultures or as natural microorganism [32]. In the same study it was also observed that conjugated linolenic acid (CLNA) produced by other LAB strains of *L. curvatus* and *L. sakei* with 60.1% and 22.4% conversion respectively [32]. For *L. curvatus* and *L. sakei* strains, a poor conversion percentage of LA into CLA was reported in MRS medium in contrast to high conversion of LNA into CLNA [32]. The presence of CLA producing *L. salivarius* was observed in a diverse group of samples. *L. salivarius* have been isolated from animal origin [33] and applied as the direct fed microbial starter for the early development of calf rumen [34]. Ting et al [35] characterized CLA producing LAB as the potential probiotic for chicken. Dahiya and Puniya [13] reported *L. salivarius* isolated from dairy based products and infant fecal origin and showed to produce CLA less than 25 μg/mL. Isolate CCB1 (*L. salivarius* strain P2) isolated from chicken was observed to produce 21.97 μg/mL of CLA after 48 h conversion [35]. Although the production of CLA in our study is little higher, it is defensible as the production is highly strain specific [26]. The isolates of *L. ingluviei* are a group of LAB studied in the gastrointestinal tracts of birds [36] however their presence in goat rumen fluid it is limited to our study only. The CLA production and its effects are so far not reported from any other sources to the best of our knowledge.

## CONCLUSION

The study showed that lactobacilli isolated from goat rumen fluid have the potential to produces bioactive isomers of CLA. These isolates can be applied as direct fed microbial as they have generally recognized as safe status and easier to handle than other known CLA producers in the rumen. The isolates can be further studied for their probiotic potential and may be applied as ruminant feed additives to enhance the nutraceutical value of milk. However, more studies are required to validate these finding in suitable animal trials and their isomer specific health benefits.

## Figures and Tables

**Table 1:** Biochemical characteristics and identification of the conjugated linoleic acid positive isolates

Isolates	Catalase/Oxidase	Xylose fermentation	Melibiose fermentation	Arginine hydrolysis	Identified based on sequencing and biochemical tests
GI-15	−	ND	ND	ND	*Lactobacillus ingluviei*
GI-19	−	ND	ND	ND	*Lactobacillus salivarius*
GI-21	−	ND	ND	ND	*Lactobacillus salivarius*
GI-23	−	ND	ND	ND	*Lactobacillus ingluviei*
GI-35	−	−	−	−	*Lactobacillus curvatus*
GI-39	−	+	−	−	*Lactobacillus curvatus*
GI-40	−	−	−	−	*Lactobacillus curvatus*
GI-41	−	−	−	−	*Lactobacillus curvatus*
GI-42	−	+	+	+	*Lactobacillus sakei*
GI-43	−	−	−	−	*Lactobacillus curvatus*
GI-44	−	−	−	−	*Lactobacillus curvatus*
GI-45	−	−	−	−	*Lactobacillus curvatus*
GI-46	−	+	+	+	*Lactobacillus sakei*
GI-47	−	−	−	−	*Lactobacillus curvatus*
GI-48	−	+	−	−	*Lactobacillus curvatus*
GI-49	−	−	−	−	*Lactobacillus curvatus*
GI-51	−	+	−	−	*Lactobacillus curvatus*
GI-52	−	−	−	−	*Lactobacillus curvatus*
GI-54	−	+	+	+	*Lactobacillus sakei*
GI-55	−	−	−	−	*Lactobacillus curvatus*
GI-56	−	+	−	−	*Lactobacillus curvatus*
GI-57	−	−	−	−	*Lactobacillus curvatus*
GI-59	−	+	+	+	*Lactobacillus sakei*

GI, goat isolates; +, positive; −, negative; ND, not determined.
